# Type 2 Myocardial Infarction Caused by Orthostatic Hypotension With Post-transcatheter Aortic Valve Implantation: A Case Report

**DOI:** 10.7759/cureus.53921

**Published:** 2024-02-09

**Authors:** Hitomi Tsuchida, Shiho Amano, Chiaki Sano, Ryuichi Ohta

**Affiliations:** 1 Family Medicine, Japanese Red Cross Society Himeji Hospital, Himeji, JPN; 2 Community Care, Unnan City Hospital, Unnan, JPN; 3 Community Medicine Management, Shimane University Faculty of Medicine, Izumo, JPN

**Keywords:** left ventricular outflow tract stenosis, orthostatic hypotension, general medicine, rural health services, elderly, type2 myocardial infarction, transcatheter aortic valve implantation

## Abstract

This case report delineates the occurrence and management of type 2 myocardial infarction (MI) in an 89-year-old woman following transcatheter aortic valve implantation (TAVI). The patient, with a history of severe aortic stenosis, hypertension, dyslipidemia, and colorectal cancer, presented with nausea and significant hypotension. Initial assessments revealed elevated troponin levels, atrial fibrillation, and ST-segment depression, leading to a diagnosis of type 2 MI. This condition was attributed to the interplay between left ventricular hypertrophy, hypotension-induced dehydration, and increased myocardial oxygen demand. The patient with post-TAVI exhibited dynamic changes in cardiac hemodynamics, with improvements in left ventricular function but persistent hypertrophy and diastolic dysfunction. This state, combined with hypotension due to diuretic-induced dehydration and atrial fibrillation, precipitated a mismatch in myocardial oxygen supply and demand. The cessation of diuretics and initiation of rehydration therapy stabilized her condition, with subsequent normalization of troponin levels and blood pressure. This case highlights the complexity of managing type 2 MI in elderly patients post-TAVI. It underscores the importance of holistic consideration of both myocardial oxygen supply and demand factors, particularly in left ventricular hypertrophy and diastolic dysfunction. The multifactorial nature of type 2 MI necessitates a tailored approach to diagnosis and management, emphasizing the need for comprehensive post-procedural care in patients undergoing TAVI.

## Introduction

In aging populations of developed countries, the number of type 2 myocardial infarctions has increased, necessitating varied case management. Myocardial infarctions (MI) are classified into five types, predominantly feature type 1, caused by coronary artery plaque changes, and type 2, resulting from an oxygen supply-demand mismatch in the myocardium [1]. Conditions like sepsis, anemia, arrhythmia, hypotension, and severe aortic stenosis can precipitate type 2 infarctions [2]. Severe aortic stenosis often leads to left ventricular hypertrophy, reducing myocardial capillary density and increasing oxygen demand [3]. This hypertrophy is implicated in myocardial ischemia in patients with non-obstructive coronary artery disease [4]. Type 2 MI usually presents mild symptoms, less noticeable electrocardiographic changes, and lower troponin levels than type 1 [5]. Diagnosis hinges on elevated cardiac troponin levels and an oxygen supply-demand imbalance unrelated to atherothrombosis [6]. Mortality risk post-event is comparable between types 1 and 2 [7]. Treatment depends on the underlying cause [5]. This case highlights a type 2 MI in an elderly woman with post-transcatheter aortic valve implantation (TAVI), attributed to left ventricular hypertrophy and hypotension-induced dehydration, underscoring the importance of tailored management for such patients.

## Case presentation

Patient background 

An 89-year-old woman was admitted to a rural community hospital due to pronounced symptoms of nausea and a significant decrease in blood pressure. On the day she visited the hospital, she experienced acute nausea and was unable to stand. Her systolic blood pressure was recorded at 90 mmHg, a notable drop from her usual 120 mmHg. As her symptoms persisted, she was urgently transferred to the emergency department.

Her medical history was notable for TAVI performed for severe aortic stenosis a year ago. Additionally, she had a history of heart failure, hypertension, dyslipidemia, and colorectal cancer, for which she had undergone surgery. Her current medications included sacubitril-valsartan sodium (150 mg), rosuvastatin calcium (2.5 mg), donepezil (5 mg), empagliflozin (10 mg), aspirin (100 mg), spironolactone (25 mg), bonoprazan (10 mg), and tolvaptan (7.5 mg).

Initial assessment 

Upon physical examination, her vital signs were as follows: blood pressure at 92/45 mmHg, pulse rate at 130 beats/min, body temperature at 36.2°C, respiratory rate at 24 breaths/min, and an oxygen saturation of 99% on room air. The patient was coherent and alert. A cardiac examination revealed irregular heart sounds without any murmur and bilateral late crackles in the lungs without edema in the lower legs. However, the extremities were slightly cold. Laboratory findings indicated elevated cardiac markers, notably Troponin I (Table 1).

**Table 1:** Initial laboratory data of the patient CRP, C-reactive protein

Parameter	Level	Reference
White blood cells	9.3	3.5–9.1 × 10^3^/μL
Neutrophils	80.8	44.0–72.0%
Lymphocytes	10.0	18.0–59.0%
Hemoglobin	11.2	11.3–15.2 g/dL
Hematocrit	33.5	33.4–44.9%
Mean corpuscular volume	97.2	79.0–100.0 fl
Platelets	16.6	13.0–36.9 × 10^4^/μL
Total protein	7.3	6.5–8.3 g/dL
Albumin	3.9	3.8–5.3 g/dL
Total bilirubin	0.6	0.2–1.2 mg/dL
Aspartate aminotransferase	30	8–38 IU/L
Alanine aminotransferase	12	4–43 IU/L
Lactate dehydrogenase	326	121–245 U/L
Blood urea nitrogen	32.7	8–20 mg/dL
Creatinine	1.88	0.40–1.10 mg/dL
Serum Na	143	135–150 mEq/L
Serum K	4.9	3.5–5.3 mEq/L
Serum Cl	108	98–110 mEq/L
CRP	0.05	<0.30 mg/dL
Troponin I	1.064	<0.02 ng/mL
Urine test		
Leukocyte	1+	-
Protein	1+	-
Blood	2+	-

The electrocardiogram exhibited atrial fibrillation with an ST-segment depression in leads V4 and V5. Post the administration of 500 ml of extracellular fluid, her blood pressure improved to 101/61 mmHg and her heart rhythm normalized to sinus rhythm.

**Figure 1:**
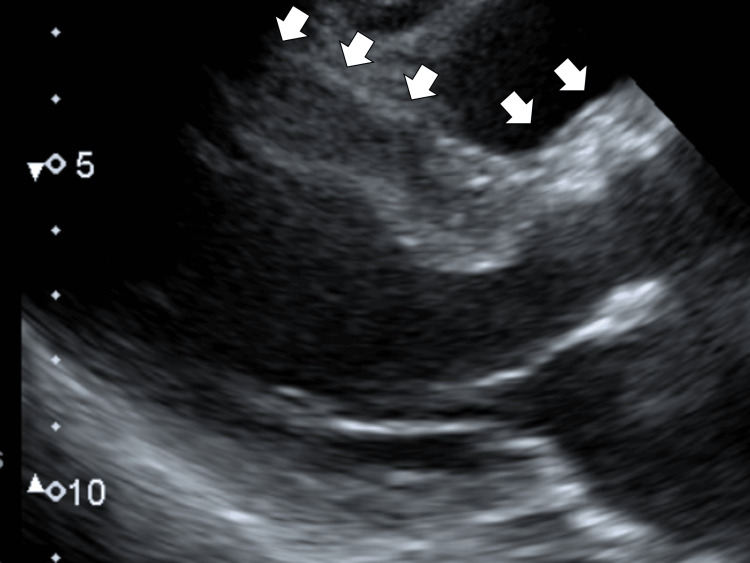
Initial echocardiography showing left ventricular septum thickening without the narrowing aortic valve region (white arrows)

An echocardiographic evaluation revealed basal area hypokinesis but no significant asynergy. The left ventricular septum was thickened, measuring 13-14 mm (Figure 1).

**Figure 2:**
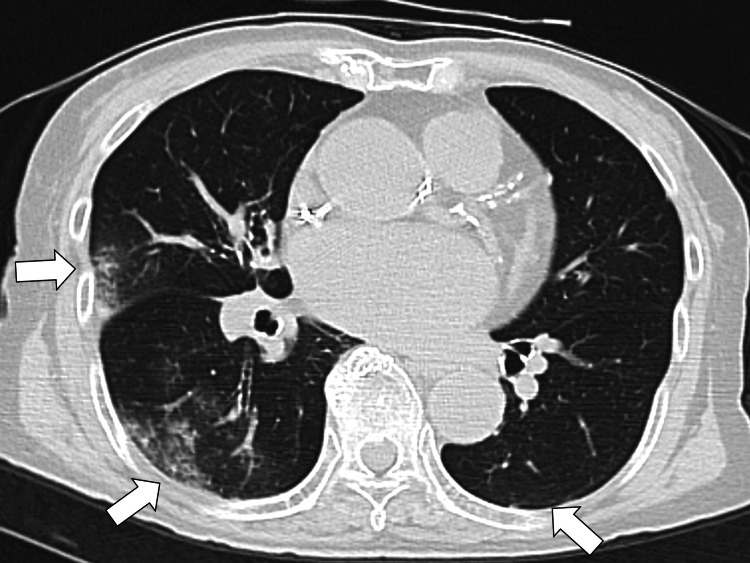
Chest computed tomography showing bilateral lung congestion (white arrows)

Diastolic dysfunction was evident with an E/e' of 26.6 ml/m^2^ in the ventricular septal wall (normal range: 2.6-17) and 17.7 ml/m^2^ in the lateral wall (normal range: 2.6-17). The left atrial volume index (LAVI) was 168 ml/m^2 ^(normal range: ≤28 mL/m^2^), and the tricuspid regurgitant velocity (TRV) was 3.0 m/s (normal range: ≤2.5 m/s). A chest X-ray and computed tomography showed bilateral lung congestion (Figure 2).

Diagnosis 

The clinical presentation, alongside these findings, led to a diagnosis of type 2 myocardial infarction. This condition was attributed to reduced coronary return resulting from left ventricular hypertrophy, impaired left ventricular dilation, and hypotension due to dehydration. Contributing factors included increased oxygen demand due to atrial fibrillation and a reduced oxygen supply owing to hypotension, exacerbated by left ventricular outflow tract stenosis.

Treatment and outcome 

On her second day of admission, diuretics, identified as a potential cause of her dehydration, were discontinued. Rehydration therapy was initiated, resulting in her blood pressure stabilizing at 117/54 mmHg. Troponin levels were initially elevated to 65.35 ng/ml and reduced to 45.35 ng/ml. Her blood pressure remained stable throughout her hospital stay, and her daily activities returned to pre-admission levels. Her troponin levels became normal, and she was discharged on the twenty-fifth day of her hospitalization.

## Discussion

In this study, we report a case of a patient who underwent TAVI and subsequently exhibited elevated troponin levels. Notably, the patient's electrocardiography and echocardiography results did not indicate any apparent cardiovascular compromise, leading to the consideration of type 2 MI as a likely diagnosis. This case report delves into the causative factors of type 2 MI and the alterations in myocardial hemodynamics post-TAVI.

A discrepancy between heightened myocardial oxygen demand and a reduced oxygen supply characterizes type 2 MI. Factors influencing oxygen demand encompass systolic wall stress, myocardial contractility, and heart rate [8]. In the discussed case, augmented myocardial contractility, attributed to left ventricular hypertrophy and an increased heart rate due to atrial fibrillation were probable contributing factors [9]. Meanwhile, factors leading to diminished supply include compromised coronary vascular blood flow and oxygen delivery capabilities, which can be caused by coronary artery spasm, embolism, vascular endothelial dysfunction, hypotension, and anemia, as the same pathophysiology of aortic dissection [10-12].

For the patient in question, hypotension was a consequence of reduced fluid intake and circulating plasma, the latter being a result of intravascular dehydration induced by diuretic therapy for pre-existing congestive heart failure before TAVI. Additionally, a decline in cardiac output due to atrial fibrillation was observed. Furthermore, the constriction of the left ventricular outflow tract, explored in subsequent sections, may have also played a role in diminishing cardiac output and oxygen supply.

Post-TAVI cardiac alterations typically include relief of aortic valve stenosis, prompt normalization of afterload, and consequent improvements in left ventricular volume, remodeling, systolic and diastolic function, and ejection fraction [13]. However, a swift decrease in afterload may induce new dynamic intraventricular gradients and stenosis of the left ventricular outflow tract [13]. Predominant risk factors for these gradients include a small left ventricular end-diastolic diameter, elevated ejection fraction, significant valve pressure gradient, and limited left ventricular volume [14].

In the present case, the patient exhibited a higher ejection fraction due to reduced afterload and a small end-diastolic diameter (34 mm). Persistent left ventricular wall hypertrophy with a ventricular septal wall thickness of 14 mm was noted. Additionally, LAVI was 68.7 ml/m2, E/e' ratios were 26.6 (septum) and 17 (lateral), and TRV was 3.0 m/s. These findings indicated ventricular dilation defects and aligned with the aforementioned risk factors. Therefore, stenosis of the left ventricular outflow tract may have led to reduced cardiac output, culminating in a type 2 MI. Previous research has demonstrated that approximately 10% of patients experience at least one acute coronary syndrome episode within an average of 25 months post-TAVI, with type 2 MI being the most frequent, accounting for 35.9% of cases [15]. This prevalence further underscores the significance of the discussed condition as a contributing factor to type 2 MI in post-TAVI patients.

## Conclusions

This case exemplifies the multifaceted etiology of type 2 myocardial infarctions. Type 2 MI can arise from various mechanisms but is particularly prevalent in patients with residual left ventricular hypertrophy and diastolic dysfunction post-TAVI. Therefore, vigilant management of additional factors that could elevate myocardial oxygen demand or curtail supply is imperative.

## References

[1] Merlo AC, Bona RD, Ameri P, Porto I (2022). Type 2 myocardial infarction: a diagnostic and therapeutic challenge in contemporary cardiology. Intern Emerg Med.

[2] Pillai B, Trikkur S, Farooque U (2020). Type II myocardial infarction: predisposing factors, precipitating elements, and outcomes. Cureus.

[3] Bache RJ, Dai XZ (1990). Myocardial oxygen consumption during exercise in the presence of left ventricular hypertrophy secondary to supravalvular aortic stenosis. J Am Coll Cardiol.

[4] Eskerud I, Gerdts E, Larsen TH, Lønnebakken MT (2019). Left ventricular hypertrophy contributes to Myocardial Ischemia in Non-obstructive Coronary Artery Disease (the MicroCAD study). Int J Cardiol.

[5] Rafiudeen R, Barlis P, White HD, van Gaal W (2022). Type 2 MI and myocardial injury in the era of high-sensitivity troponin. Eur Cardiol.

[6] Thygesen K, Alpert JS, Jaffe AS, Chaitman BR, Bax JJ, Morrow DA, White HD (2018). Fourth universal definition of myocardial infarction (2018). J Am Coll Cardiol.

[7] Chapman AR, Shah AS, Lee KK (2018). Long-term outcomes in patients with type 2 myocardial infarction and myocardial injury. Circulation.

[8] McCarthy CP, Kolte D, Kennedy KF, Vaduganathan M, Wasfy JH, Januzzi JL Jr (2021). Patient Characteristics and Clinical Outcomes of Type 1 Versus Type 2 Myocardial Infarction. J Am Coll Cardiol.

[9] Belkouche A, Yao H, Putot A (2021). The multifaceted interplay between atrial fibrillation and myocardial infarction: a review. J Clin Med.

[10] Abe T, Samuel I, Eferoro E, Samuel AO, Monday IT, Olunu E, Fakoya AO (2021). The diagnostic challenges associated with type 2 myocardial infarction. Int J Appl Basic Med Res.

[11] Ohta R, Sano C (2023). Aortic dissection and hypotension without cardiac tamponade: a case report. Cureus.

[12] Muhammad R, Lefi A, Ghassani DN, Mulia EP (2022). An atypical presentation of aortic dissection: echocardiography for accurate detection. J Ultrasound.

[13] Graziani F, Cialdella P, Lillo R (2022). Acute haemodynamic impact of transcatheter aortic valve implantation in patients with severe aortic stenosis. ESC Heart Fail.

[14] Olsen KR, LaGrew JE, Awoniyi CA, Goldstein JC (2018). Undiagnosed hypertrophic obstructive cardiomyopathy during transcatheter aortic valve replacement: a case report. J Med Case Rep.

[15] Vilalta V, Asmarats L, Ferreira-Neto AN (2018). Incidence, clinical characteristics, and impact of acute coronary syndrome following transcatheter aortic valve replacement. JACC Cardiovasc Interv.

